# Pain Outcomes in Patients with Metastatic Castration-Resistant Prostate Cancer Treated with ^223^Ra: PARABO, a Prospective, Noninterventional Study

**DOI:** 10.2967/jnumed.123.265557

**Published:** 2023-09

**Authors:** Holger Palmedo, Hojjat Ahmadzadehfar, Susanne Eschmann, Andreas Niesen, Johann Schönberger, Vahé Barsegian, Knut Liepe, Felix M. Mottaghy, Rongjin Guan, Joerg Pinkert, Per Sandström, Ken Herrmann

**Affiliations:** 1 Institute of Radiology and Nuclear Medicine Kaiser Passage and PET/CT Centre, Johanniter Hospital, Bonn, Germany;; 2 Klinikum Westfalen and MVZ Prof. Uhlenbrock and Partner, Dortmund, Germany;; 3 Marienhospital Stuttgart, Stuttgart, Germany;; 4 Diakovere Henriettenstift, Hannover, Germany;; 5 Klinikum Weiden, Weiden, Germany;; 6 Helios Kliniken Schwerin, Schwerin, Germany;; 7 Department of Nuclear Medicine, Klinikum Frankfurt (Oder) GmbH, Frankfurt, Germany;; 8 Department of Nuclear Medicine, University Hospital RWTH Aachen University, Aachen, Germany, and Department of Radiology and Nuclear Medicine, Maastricht University Medical Center, Maastricht, The Netherlands;; 9 Bayer HealthCare Pharmaceuticals, Whippany, New Jersey;; 10 Bayer AG, Berlin, Germany; and; 11 Department of Nuclear Medicine, University of Duisburg–Essen, and German Cancer Consortium–University Hospital Essen, Essen, Germany

**Keywords:** targeted α-therapy, ^223^Ra, castration-resistant prostate cancer, bone metastases, pain response

## Abstract

^223^Ra, a targeted α-therapy, is approved for the treatment of patients with metastatic castration-resistant prostate cancer (mCRPC) who have bone metastases. In the phase 3 ALSYMPCA study, ^223^Ra prolonged survival and improved quality of life versus placebo. Our real-world study, PARABO, investigated pain and bone pain–related quality of life in patients with mCRPC and symptomatic bone metastases receiving ^223^Ra in clinical practice. **Methods:** PARABO was a prospective, observational, noninterventional single-arm study conducted in nuclear medicine centers across Germany (NCT02398526). The primary endpoint was a clinically meaningful pain response (≥2-point improvement from baseline for the worst-pain item score in the Brief Pain Inventory–Short Form). **Results:** The analysis included 354 patients, who received a median of 6 ^223^Ra injections (range, 1–6). Sixty-seven percent (236/354) received 5–6 injections, and 33% (118/354) received 1–4 injections. Of 216 patients with a baseline worst-pain score of more than 1, 59% (128) had a clinically meaningful pain response during treatment. Corresponding rates were 67% (range, 98/146) with 5–6 ^223^Ra injections versus 43% (range, 30/70) with 1–4 injections, 60% (range, 60/100) in patients with no more than 20 lesions versus 59% (range, 65/111) in those with more than 20 lesions, and 65% (range, 69/106) in patients without prior or concomitant opioid use versus 54% (range, 59/110) in those with prior or concomitant opioid use. Mean subscale scores (pain severity and pain interference) on the Brief Pain Inventory–Short Form improved during treatment. **Conclusion:** ^223^Ra reduced pain in patients with mCRPC and symptomatic bone metastases, particularly in patients who received 5–6 injections. The extent of metastatic disease did not impact pain response.

Most patients with advanced prostate cancer develop bone metastases (1). The formation and growth of such metastatic lesions lead to bone pain, which is distressing for patients (2). The pathophysiology of bone pain in metastatic prostate cancer is complex, with skeleton-related events, including pathologic fractures, spinal cord compression, hypercalcemia, and neurologic deficits, playing a significant role (3). Although more than 50% of patients with bone metastases present with skeletal complications, some experience pain not associated with fractures or nerve compression (4). Chronic pain syndrome is an important complication of bone metastases and negatively impacts overall survival (OS) and quality of life (QoL) (5).

Currently, pharmacologic management of bone pain in patients with bone metastases involves mainly nonsteroidal antiinflammatory drugs and opioid analgesics used with different adjuvant therapies (6), according to the World Health Organization analgesic ladder framework (7). Nonpharmacologic management of bone pain in this setting involves palliation using external-beam radiotherapy (8).

^223^Ra-dichloride (^223^Ra) is an α-particle–emitting radiopharmaceutical approved for the treatment of patients with bone-dominant metastatic castration-resistant prostate cancer (mCRPC), based on the registrational phase 3 ALSYMPCA study (9). In this study, ^223^Ra prolonged OS (median, 14.9 vs 11.3 mo; hazard ratio, 0.70; *P* < 0.001) and the time to the first symptomatic skeletal event (SSE) (median, 15.6 vs 9.8 mo; hazard ratio, 0.66; *P* < 0.001) versus placebo, when each was used in combination with the standard of care (9). ^223^Ra also prolonged the time to external-beam radiotherapy for bone pain, reduced the risk of spinal cord compression, and improved QoL versus placebo (10*,*^11^). The short- and long-term safety profiles of ^223^Ra were favorable compared with those of placebo, with low myelosuppression rates (9*,*^12^).

ALSYMPCA was conducted on a well-defined patient population (9), and real-world studies of ^223^Ra in mCRPC reporting on pain were limited in size or were not specifically designed to assess pain (13–16). Furthermore, since ALSYMPCA, several anticancer agents, such as abiraterone, enzalutamide, and cabazitaxel, have been approved and introduced into routine clinical management for mCRPC. Therefore, a more robust evaluation of the effects of ^223^Ra on bone pain in patients with mCRPC in a real-world setting is warranted.

The PARABO study (NCT02398526) was designed to investigate the effect of ^223^Ra on pain and bone pain–related QoL in patients with mCRPC and symptomatic bone metastases in routine clinical practice. Here, we report the pain response, bone pain–related QoL, OS, and safety outcomes of ^223^Ra in PARABO.

## MATERIALS AND METHODS

### Study Design and Patients

PARABO was an observational, prospective single-arm cohort study designed to assess pain and bone pain–related QoL in patients with mCRPC receiving ^223^Ra across nuclear medicine centers in Germany. The study design is depicted in Supplemental Figure 1 (supplemental materials are available at http://jnm.snmjournals.org). Eligible male patients had a diagnosis of mCRPC with symptomatic bone metastases and no known visceral metastases. Treatment with ^223^Ra was initiated as per each investigator’s routine clinical practice. Patients participating in an investigational program with interventions outside routine clinical practice or participating in another observational study with ^223^Ra were excluded. Documented approval from appropriate independent ethics committees or institutional review boards was obtained for all participating sites before the study. All patients provided written informed consent before study participation.

### Treatment Schedule

^223^Ra was administered at a 55-kBq (1.485 μCi)/kg dose by intravenous injection every 4 wk, up to a maximum of 6 injections.

### Endpoints

The primary endpoint was a clinically meaningful pain response, defined as an improvement by at least 2 points from baseline in the worst-pain item of the Brief Pain Inventory–Short Form at any postbaseline assessment. Secondary endpoints included change in pain- and bone pain–related QoL over time during ^223^Ra treatment, pain control and progression rates, time to first pain progression, time to first opioid use, covariates of pain response during treatment, pain response based on extent of bone metastases at baseline, OS, treatment-emergent adverse events, number of fractures, and time to first SSE. QoL was assessed using a questionnaire: Functional Assessment of Cancer Therapy Quality-of-Life Measurement in Patients with Bone Pain. All secondary endpoints are detailed in the supplemental methods.

### Data Sources

Investigators collected historical data (demographic and clinical characteristics) from medical records. Treatment-related data, the results of tumor assessments, and other disease status information were collected during routine practice visits. For patient-reported outcomes, questionnaires were completed by patients during routine visits.

### Statistical Considerations

For sample size calculation, the precision of the estimate for the primary outcome (pain response rate) was considered. Precision was defined by the width of the 95% CI of the estimate with a given sample size. A precision of less than 20% was considered clinically meaningful, taking the variance of pain measurements into account. Assuming that at least 60% of patients would be evaluable for the primary analysis of pain response at a postbaseline assessment and that 30%–70% of these evaluable patients would show a pain response, at least 350 patients would be required to achieve a precision of less than 20%. Sample size calculations were performed with the nQuery 7 (Statsols) platform. Statistical analyses in this study were primarily of an explorative and descriptive nature.

## RESULTS

### Baseline Characteristics

Between March 2015 and December 2017, 358 patients were enrolled at 27 medical centers; 356 patients received at least 1 dose of ^223^Ra. Two patients who did not meet all eligibility criteria were excluded; thus, 354 patients were included in the full analysis set. Patient enrollment, treatment, and eligibility are shown in Supplemental Figure 2. Patient baseline characteristics are given in Table 1. Overall, 58% (204/354), 17% (61/354), and 8% (27/354) of patients had an Eastern Cooperative Oncology Group performance status of 1, 2 or 3–4, respectively; 36% (127/354) had more than 20 bone metastases; and 69% (243/354) had mild pain according to the World Health Organization pain ladder. Sixty-two percent (219/354) of patients had received at least 1 prior systemic anticancer medication, with docetaxel being the most common (34% [119/354]). Before the study, 13% (45/354) of patients used at least 1 bone health agent, and 33% (116/354) used opioids.

**TABLE 1.** Baseline Characteristics (Full Analysis Set, *n* = 354)

Characteristic	Data	Characteristic	Data
Age (y)	74 (43–91)	World Health Organization pain ladder	
EOG performance status		Step 1 (mild pain)	243 (69%)
0	56 (16%)	Step 2 (moderate pain)	74 (21%)
1	204 (58%)	Step 3 (severe pain)	37 (10%)
2	61 (17%)	Prior bone health agents	
3–4	27 (8%)	≥1 medication	45 (13%)
Missing	6 (2%)	Denosumab	19 (5%)
Months from diagnosis to initial visit	55 (2–321)	Zoledronic acid	25 (7%)
Months from bone metastases to initial visit	28 (0–243)	Bisphosphonates	1 (<1%)
Months from castration resistance to initial visit	10 (0–155)	Prior radiotherapy	194 (55%)
ALP (U/L)		Prior LPTs that ended before start of ^223^Ra*	
Median	133.0	≥1	219 (62%)
<150	134 (38%)	Docetaxel	119 (34%)
150–300	52 (15%)	Cabazitaxel	29 (8%)
>300	44 (12%)	Abiraterone	83 (23%)
Missing	124 (35%)	Enzalutamide	51 (14%)
PSA (μg/L)		Prior LPTs, including those overlapping ^223^Ra treatment^†^	
Median	58.0	0	112 (32%)
<50	118 (33%)	1	119 (34%)
50–200	70 (20%)	2	65 (18%)
>200	61 (17%)	≥3	58 (16%)
Missing	105 (30%)	Opioid use before or at baseline	116 (33%)
Extent of bone disease (*n* = 335)			
<6 metastases	37 (10%)		
6–20 metastases	124 (35%)		
>20 metastases	127 (36%)		
Superscan	55 (16%)		
Missing	9 (3%)		

* Selected prior LPTs are shown.

† Abiraterone, enzalutamide, cabazitaxel, and docetaxel.

ECOG = Eastern Cooperative Oncology Group; LPT = life-prolonging therapy.

Qualitative data are number and percentage; continuous data are median with or without range.

Baseline characteristics for patients who received 1–4 or 5–6 ^223^Ra injections are shown in Supplemental Table 1. Among patients with an Eastern Cooperative Oncology Group performance status of 2–4, a superscan, opioid use (World Health Organization pain level, 2–3), an alkaline phosphatase (ALP) level of more than 300 U/L, a prostate-specific antigen (PSA) level of more than 200 μg/L, or at least 3 prior systemic anticancer therapies, a greater proportion received 1–4 than 5–6 injections (Supplemental Table 1).

### Treatments

The median number of ^223^Ra injections received was 6 (range, 1–6); 33% (118/354) of patients received 1–4 injections, and 67% (236/354) received 5–6 injections (Supplemental Table 2). ^223^Ra treatment was delayed or interrupted in 6% (23/354) of patients, most often for adverse events (Supplemental Table 2). Adverse events were also the most common reason for ^223^Ra discontinuation (12%), followed by progression of underlying disease (10%), the patient’s decision (8%), and death (6%).

The likelihood of receiving a higher number of ^223^Ra injections was greatest for patients who received concomitant enzalutamide and lowest for patients with a higher number of bone metastases, higher baseline ALP or PSA levels, opioid use, or prior chemotherapy (Supplemental Table 3). The percentage of patients with blood cell counts below the limit for further injections at each treatment visit is shown in Supplemental Table 4. The median time from castration resistance to the first ^223^Ra injection was 10 mo (range, 0–155 mo) (Table 1). The median time to the next treatment with a life-prolonging therapy was 12.1 mo (95% CI, 7.2–not reached [NR]) in patients who received 1–4 ^223^Ra injections and 21.8 mo (95% CI, 14.8–NR) in those who received 5–6 injections (Supplemental Fig. 3).

### Pain Outcomes

**FIGURE 1.**
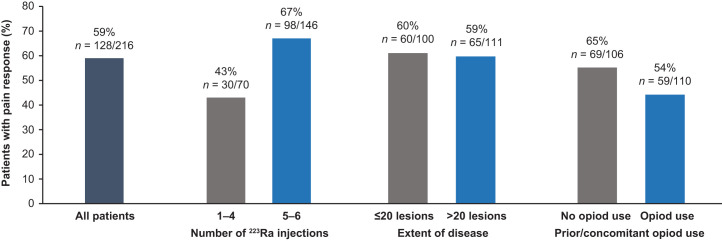
Rates of clinically meaningful pain response. Responses were evaluated in patients with baseline Brief Pain Inventory–Short Form worst-pain item score > 1 (*n* = 216), with data also stratified by number of ^223^Ra injections, disease extent, and prior or concomitant opioid use.

A clinically meaningful pain response occurred in 59% (128/216) of evaluable patients overall. Among the individual subgroups assessed, rates of clinically meaningful pain response were 1.6-fold greater in patients who received 5–6 than 1–4 ^223^Ra injections, 1.2-fold greater in patients without than with prior or concomitant opioid use, and generally similar regardless of lesion number (Fig. 1).

When the effects of covariates on this pain response were assessed, greater rates of pain response were seen at most treatment visits in patients with a baseline ALP of more than 300 U/L than in those with less than 150 or 150 − 300 U/L (Supplemental Table 5), patients with baseline PSA of less than 50 or 50–200 μg/L than in those with more than 200 μg/L (Supplemental Table 6), and patients who were taking nonopioid analgesics or weak opioids than those taking strong opioids (Supplemental Table 7). No other covariates had consistent effects on response rates, including the number of known bone metastases at baseline or prior treatment with chemotherapy, abiraterone, enzalutamide, or bone health agents.

**FIGURE 2.**
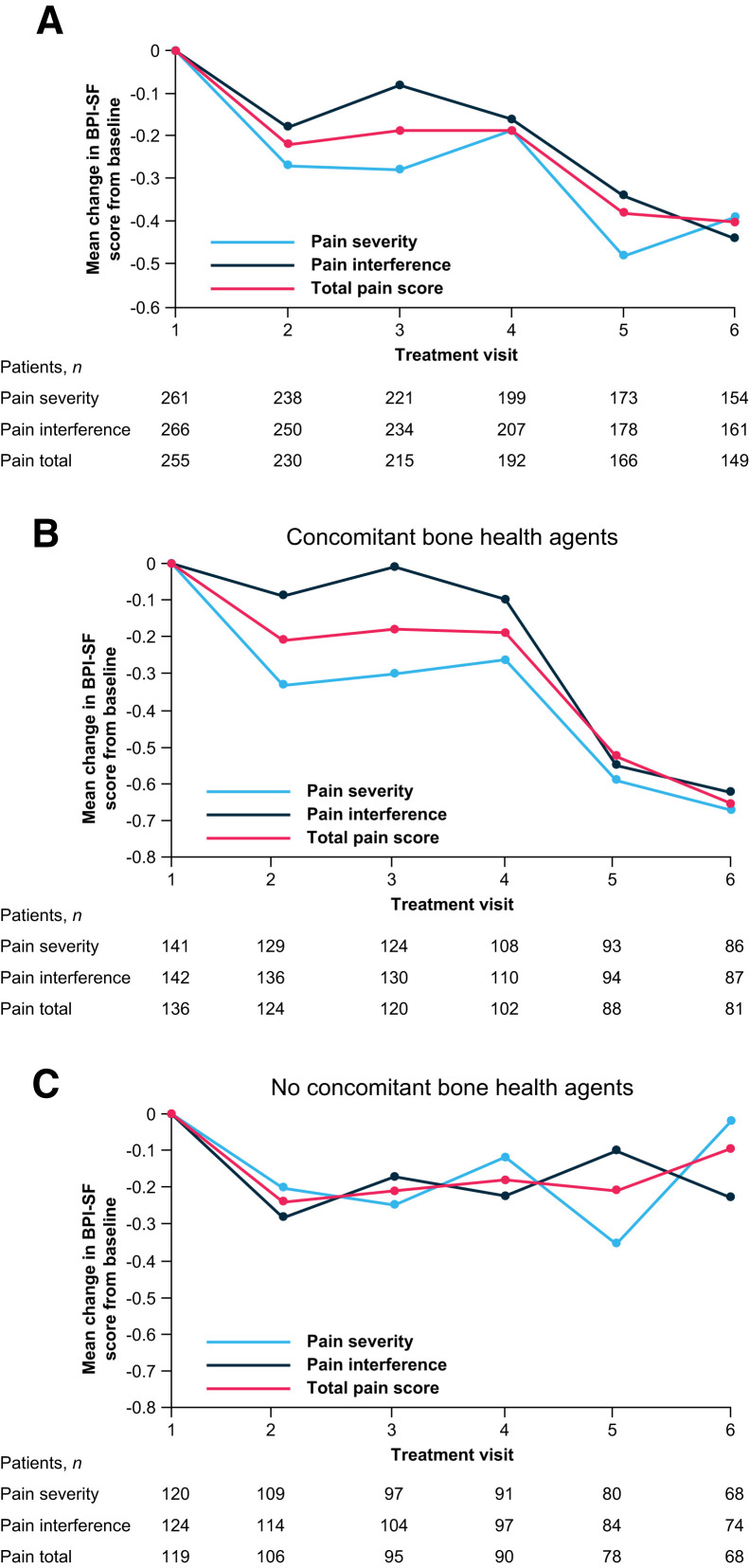
Changes in pain over time. Treatment visits correspond to ^223^Ra injections. Shown are mean change in mean Brief Pain Inventory–Short Form scores from baseline (full analysis set; 274/354 patients completed Brief Pain Inventory–Short Form questionnaire and were included). BPI-SF = Brief Pain Inventory–Short Form.

**FIGURE 3.**
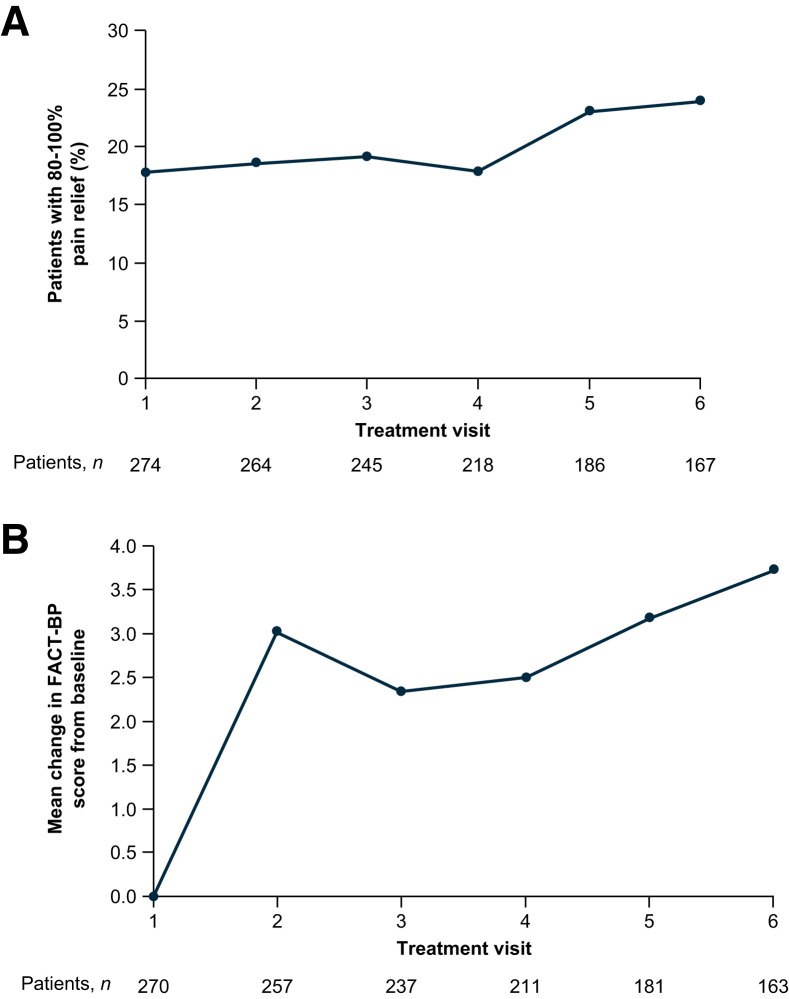
Changes in proportion of patients with complete or nearly complete pain relief and bone pain–related QoL over time. Treatment visits correspond to ^223^Ra injections. (A) Proportion of patients with 80%−100% pain relief. (B) Mean change from baseline on scores for Functional Assessment of Cancer Therapy Quality-of-Life Measurement in Patients with Bone Pain. FACT-BP = Functional Assessment of Cancer Therapy Quality-of-Life Measurement in Patients with Bone Pain.

The total pain score and pain severity and interference subscale scores on the Mean Brief Pain Inventory–Short Form improved from baseline during ^223^Ra treatment (Fig. 2A). Improvements from baseline in these scores were notable in patients who received concomitant bone health agents (Fig. 2B), whereas no clear benefit was seen in patients without concomitant bone health agents (Fig. 2C). Of patients who received 6 ^223^Ra injections, 24% (40/167) reported complete or nearly complete (80%–100%) pain relief (Fig. 3A). Pain control was reported in 67% (145/216) of patients, and pain progression was reported in 33% (71/216). Mean improvements from the baseline score on the Functional Assessment of Cancer Therapy Quality-of-Life Measurement in Patients with Bone Pain (indicating improved bone pain–related QoL) were seen from treatment visit 2 onward, with the greatest improvement seen at visit 6 (Fig. 3B).

The median time to the first pain progression was 6.70 mo (95% CI, 6.44 mo–NR); the time to pain progression was shorter in patients treated with 1–4 than 5–6 ^223^Ra injections but was not impacted by concomitant bone health agent use (Supplemental Fig. 4). The median time to the first opioid use in patients who had not received prior opioids was NR (Supplemental Fig. 5).

**FIGURE 4.**
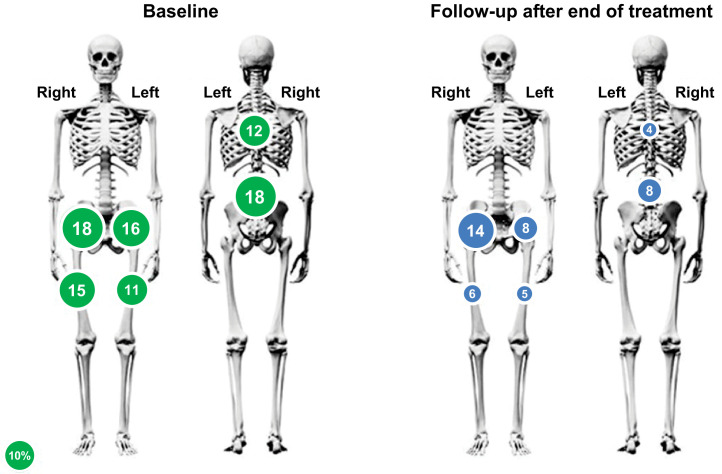
Areas that hurt most at baseline and at follow-up after end of treatment. Numbers indicate percentage of patients (≥10%) who reported areas that hurt most at baseline and end of treatment (in same areas as baseline). Patients may have reported pain in more than one area. Circles indicate pelvis (left and right), thigh (left and right), thoracic vertebrae, and lumbar vertebrae. Circle sizes represent percentage of patients who reported that area; 10% reference scale is shown.

At each study visit, the mean decrease from baseline in worst-pain scores was greater in patients with strong bone uptake than in those with weaker bone uptake (Supplemental Table 8). For all skeletal areas that were most frequently reported as hurting at baseline (lumbar and thoracic vertebrae, left and right pelvis, and left and right thigh), fewer patients reported these areas as still hurting in the posttreatment follow-up period (Fig. 4).

### OS

**FIGURE 5.**
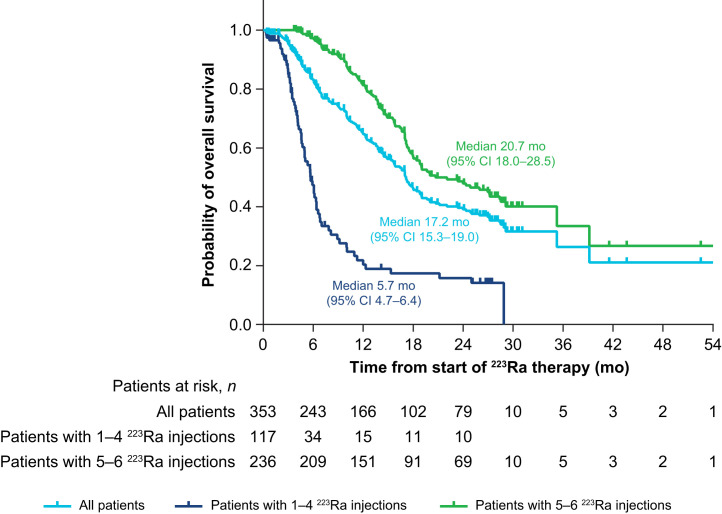
OS: full analysis set (*n* = 354).

Median OS was 17.15 mo (95% CI, 15.33–18.97 mo) in the total patient population and was longer in patients who received 5–6 ^223^Ra injections than in those who received 1–4 injections (Fig. 5). Median OS was shorter in patients who had received prior abiraterone or enzalutamide than in those who had not received these agents and was longer in patients who had received bone health agents during ^223^Ra treatment than in those who had not (Supplemental Table 9).

### Safety

Any-grade treatment-emergent adverse events occurred in 56% (200/356) of patients, and serious treatment-emergent adverse events occurred in 27% (96/356) (Supplemental Table 10). The most common treatment-emergent adverse event and drug-related treatment-emergent adverse event was anemia (13% [47/356] and 9% [33/356], respectively), whereas few patients experienced pancytopenia (2% for each) or thrombocytopenia (2% for each) (Table 2). Grade 5 treatment-emergent adverse events occurred in 8% (27/356) of patients; 1% (5/356) of patients experienced drug-related grade 5 treatment-emergent adverse events (Supplemental Table 10), which included pancytopenia (*n* = 4) and metastasis to soft tissue (*n* = 1).

**TABLE 2.** Treatment-Emergent and Drug-Related Treatment-Emergent Adverse Events Occurring in at Least 2% of Patients (Safety Analysis Set, *n* = 356)

Adverse event	*n*
Treatment-emergent	
Any	200 (56%)
Anemia	47 (13%)
Fatigue	28 (8%)
Diarrhea	18 (5%)
Nausea	16 (4%)
Metastases to liver	11 (3%)
Pain	11 (3%)
General physical health deterioration	10 (3%)
Back pain	8 (2%)
Bone pain	8 (2%)
Pancytopenia	8 (2%)
Thrombocytopenia	8 (2%)
Drug-related treatment-emergent	
Any	92 (26%)
Anemia	33 (9%)
Diarrhea	17 (5%)
Nausea	10 (3%)
Fatigue	9 (3%)
Pancytopenia	8 (2%)
Thrombocytopenia	8 (2%)

Treatment-emergent adverse events are according to system organ classes in *Medical Dictionary for Regulatory Activities* (MedDRA, version 23.0), and preferred term–worst grade is listed.

**FIGURE 6.**
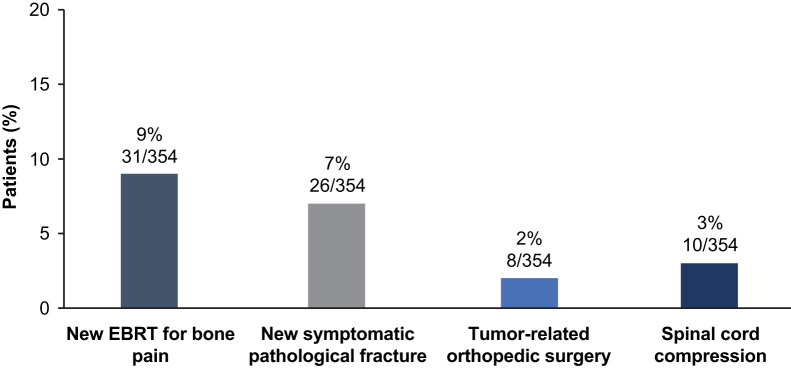
Incidence of new symptomatic skeletal events (full analysis set). EBRT = external-beam radiation therapy.

New SSEs occurred in 15% (52/354) of patients during ^223^Ra treatment and the 5-y follow-up. The most common of these events were new external-beam radiotherapy use and new symptomatic pathologic fractures (vertebral and nonvertebral) (Fig. 6). The median time to the first SSE was NR in the total patient population (95% CI, 37.45 mo–NR), in patients with 1–4 or 5–6 injections (95% CI, 24.05 mo–NR and 37.45 mo–NR, respectively), or in patients with or without concomitant bone health agent use (95% CI, 37.45–NR and NR–NR, respectively) (Supplemental Fig. 7).

The incidence rate of pathologic fractures was 0.14 (95% CI, 0.08–0.23) during the treatment period and 0.05 (95% CI, 0.03–0.09) during the 5-y follow-up period. The incidence rate of nonpathologic fractures was not recorded during treatment. However, the incidence of nonpathologic fractures during the 5-y follow-up period was 0.01 (95% CI, 0.00–0.03). The incidence proportions of pathologic fractures and bone-associated events other than fractures are shown in Supplemental Table 11. The incidence proportions of pathologic fractures and bone-associated events other than fractures were higher in recipients of 1–4 ^223^Ra injections than 5–6 injections, although only the latter had nonoverlapping 95% CIs. Patients who used concomitant bone health agents had a lower incidence of pathologic fractures and a higher incidence of bone-associated events other than fractures than did patients who did not use concomitant bone health agents, although 95% CIs were overlapping in all cases (Supplemental Table 11).

## DISCUSSION

Here, we show the real-world clinical benefit of ^223^Ra in alleviating pain and improving bone pain–related QoL in patients with mCRPC and bone metastases in the current clinical landscape. PARABO was a large (354 patients) prospective study specifically designed to assess these outcomes in a heterogeneous real-world patient population, providing strong evidence on pain response during ^223^Ra treatment relative to the other available studies in this setting, which were mostly smaller, retrospective, or not designed primarily to evaluate pain (13–16).

In our study, almost two thirds (59%) of patients experienced a clinically relevant reduction in pain during ^223^Ra treatment, consistent with the rates of pain response reported in other real-world studies (47%–58%) (13–16) and clinical trials (56%) (17) conducted on more than 30 patients. Notably, our response rate was independent of factors such as the extent of metastatic disease but was generally greater in patients with a high baseline ALP, a low baseline PSA, and no use of strong opioids, although a direct impact of opioids on bone pain is a potential confounding factor. However, improvements in pain severity, pain interference, and overall pain were notable with ^223^Ra in patients who received concomitant bone health agents but not in those who did not, suggesting that concomitant bone health agents may be beneficial for pain relief. Consistent with the pain relief associated with ^223^Ra therapy, improvements in bone pain–related QoL were also seen during treatment. The QoL benefits of ^223^Ra were also evident in a recent prospective observational study, with QoL being maintained longer in patients who completed ^223^Ra therapy (18).

Median OS was longer in this study (17.2 mo) than in ALSYMPCA (14.9 mo) (9). This finding may be related to our patients’ having less advanced disease at baseline (median ALP and PSA levels were 133 U/L and 58 μg/L, vs. 211 U/L and 146 μg/L in ALSYMPCA) or the introduction of novel anticancer treatments earlier into clinical care in the current treatment landscape. Indeed, since ALSYMPCA, there has been a shift toward using androgen receptor pathway inhibitors over chemotherapy early in the treatment sequence. How to optimally integrate ^223^Ra into the mCRPC treatment pathway continues to be an area of interest to ensure that patients receive as many life-prolonging therapies as possible. In our study, two thirds of patients received either no (32%) or 1 (34%) life-prolonging therapy before starting ^223^Ra therapy. However, the survival benefit of early- versus later-line ^223^Ra use in clinical practice is not yet clear from recent real-world studies. In the PRECISE study, slightly reduced risks of all-cause and prostate cancer–specific mortality were seen with ^223^Ra versus other mCRPC treatments, when used as a second- or third-/fourth-line treatment (19), whereas in the REACTIVATE study, second-line use of ^223^Ra was associated with longer survival than third- or later-line use (20).

Two thirds of patients in our study received 5–6 ^223^Ra injections. These patients were more likely to achieve a clinically meaningful pain response and had longer OS than those who received 1–4 injections, as is consistent with the OS findings of other real-world studies (21). Moreover, patients who completed all 6 ^223^Ra injections had the greatest improvements in bone pain–related QoL. In our study, patients who received 5–6 ^223^Ra injections had less advanced disease than those who received 1–4 injections, based on their lower ALP and PSA levels and the lower proportions of patients with an Eastern Cooperative Oncology Group performance status of 2–4, a superscan, opioid use, or at least 3 prior systemic anticancer therapies. The fact that these patients had less advanced disease may in part explain our survival findings, as such patients will have the fitness to complete 5–6 cycles of ^223^Ra.

^223^Ra had an acceptable safety profile in our study. Consistent with ALSYMPCA (9*,*^12^), rates of myelosuppression were low, with the incidence of anemia (the most common drug-related treatment-emergent adverse event), pancytopenia, and thrombocytopenia being less than 10%. Similar findings were reported with ^223^Ra in patients with mCRPC in the ongoing real-world REASSURE study (22). SSEs (the most common being new use of external-beam radiotherapy and new symptomatic pathologic fractures) occurred in a low proportion of patients during treatment with ^223^Ra and over 5 y of follow-up in our study, and there was no clear impact of bone health agents on SSEs. Notably, the incidence of pathologic fractures was consistent with the findings of the short- and long-term analyses of ALSYMPCA (9*,*^12^).

Like other real-world studies, our study had certain limitations, including the potential for suboptimal collaboration between urologists (the primary contacts for patients) and nuclear medicine physicians (involved later in the treatment course), which may affect the timeliness of ^223^Ra treatment. Additionally, it is possible for SSEs to be underreported in real-world practice for various reasons, including long intervals between patient visits, which may contribute to missing of asymptomatic fractures. However, in our study, patients were assessed regularly during the follow-up period (quarterly for the first 2 y and twice a year for the remaining 3 y).

## CONCLUSION

This large, real-world study provided evidence supporting the effectiveness of ^223^Ra in reducing pain (particularly when used in combination with bone health agents) and improving bone pain–related QoL and OS. Completion of 5–6 ^223^Ra injections was associated with the greatest benefit in these outcomes. ^223^Ra had an acceptable safety profile, consistent with that established in previous studies.

## DISCLOSURE

Holger Palmedo received sponsorship from Bayer, GE, Rotop, Sanofi-Aventis, and Curium. Hojjat Ahmadzadehfar received fees for advice or lectures from Bayer, Advanced Accelerator Applications (AAA), and SIRTEX. Andreas Niesen received fees for advice or lectures from Bayer. Felix Mottaghy is a medical advisor for NanoMab Technology Ltd. and AAA GmbH and has recently received institutional grants from NanoMab Technology Ltd., Siemens, and GE Precision Health Care LLC, all outside the submitted work. Rongjin Guan is a contractor employee at Bayer. Per Sandström is an employees of Bayer. Joerg Pinkert is an employee and stockholder of Bayer AG. Ken Herrmann reports personal fees from Bayer, SIRTEX, Adacap, Curium, Endocyte, IPSEN, Siemens Healthineers, GE Healthcare, Amgen, Novartis, and Y-mAbs; personal fees and other fees from Sofie Biosciences; nonfinancial support from ABX; and grants and personal fees from BTG, all outside the submitted work. No other potential conflict of interest relevant to this article was reported.

## Notes

KEY POINTS **QUESTION:** Does ^223^Ra alleviate pain and improve bone pain–related QoL in patients with mCRPC and symptomatic bone metastases in clinical practice? **PERTINENT FINDINGS:** PARABO, a prospective, observational real-world study, found that ^223^Ra treatment resulted in clinically meaningful pain responses in almost two thirds of patients with pain at baseline. Pain responses were more notable after 5–6 ^223^Ra injections and were not impacted by the extent of metastatic disease. **IMPLICATIONS FOR PATIENT CARE:** The findings of this large, robust study show that ^223^Ra can provide much-needed pain relief in a substantial proportion of patients with mCRPC and symptomatic bone metastases.
